# Proceedings of the Seventeenth International Society of Sports Nutrition (ISSN) Conference and Expo: Daytona Beach, FL, USA. 11-12 September 2020

**DOI:** 10.1186/s12970-020-00382-5

**Published:** 2020-11-30

## A1. Methylliberine (Dynamine™) and theacrine (TeaCrine®) magnify indices of cognitive affect when combined with coffee

### B. Raub^1^, K. Cesareo^1^, M. Carney^1^, C. Kerksick^2^, J. Sandrock^1^

#### ^1^The Center for Applied Health Sciences, Canfield, OH, 44406, USA; ^2^School of Health Sciences, Lindenwood University, MO, 63301, USA

##### **Correspondence:** B. Raub (BR@appliedhealthsciences.org)

**Background**

Methylliberine (Dynamine™) and theacrine (TeaCrine®) are purine alkaloids with pronounced neuro-energetic effects. Based on their pharmacokinetic and pharmacodynamic interaction with caffeine (i.e. threefold increase in AUC vs. caffeine alone [https://pubmed.ncbi.nlm.nih.gov/32829142]), we theorized that combining these compounds with coffee (Coffea arabica) would magnify the beneficial effects of coffee on cognitive affect.

**Methods**

This randomized, double-blind, within-subject crossover trial of 15 male (N=7) and female (N=8) subjects (mean ± age, height, weight: 32.1 ± 9.6 y, 172.9 ± 9.5 cm, 77.5 ± 19.5 kg) *assessed perceived changes in* eight indices of cognitive affect after the ingestion of coffee plus several combinations of Dynamine™ and TeaCrine®. Using a Latin Square approach to minimize potential order effects, subjects ingested five different combinations of coffee: 1) 8 oz decaffeinated coffee (DECAF), 2) 8 oz regular coffee (COF), 3) 8 oz regular coffee + 50 mg Dynamine™ (COF-D50), 4) 8 oz regular coffee + 50 mg Dynamine™ and 25 mg TeaCrine® (COF-DT), and 5) 8 oz regular coffee + 100 mg Dynamine (COF-D100) and filled out anchored visual analogue scales (VAS) that assessed *perceived changes in* mood, energy, fatigue, alertness, focus, creativity, concentration, and motivation at 0, 60, 120, and 180 min post-ingestion. Systemic hemodynamics (heart rate, blood pressure, rate pressure product, pulse pressure) were also assessed hourly during each trial. Statistical analyses (mixed factorial ANOVA and paired t-tests) were completed by an independent statistician who was blinded to treatments.

**Results**

As anticipated, DECAF only improved one VAS (focus, +9.6%, p=0.04) whereas all other treatments improved all eight VAS indices. Mixed factorial ANOVAs revealed positive changes from baseline for COF-DT in mood (+39.4%, p=0.02), energy (+70.8%, p=0.01), and motivation (+49.3%, p=0.03) and trends for improvements in fatigue (-50.4%, p=0.09), alertness (+48.5%, p=0.08), and focus (+60%, p=0.08). Compared to COF, COF-DT tended to reduce fatigue at 180 min (-32%, p=0.09). Aside from a small increase in diastolic blood pressure at 180 min (+4.3 ± 5.1 mm Hg, p=0.04) in COF-D50, no other changes in systemic hemodynamics were noted between treatments.

**Conclusions**

These findings confirm that combining Dynamine™ and TeaCrine® with caffeinated coffee significantly magnifies the beneficial effects of coffee on mood, energy, motivation while potentially reducing fatigue and improving alertness and focus. This combination was also well tolerated and had no deleterious effects on systemic hemodynamics. Future research should corroborate these findings in a larger sample size and concurrently measure serum methylxanthine and methylurate concentrations.

**Acknowledgements**

This study was funded in part by a research grant from Compound Solutions, Inc. The researchers in this study independently collected, analyzed, and interpreted the results without input from the sponsor. All authors declare no conflict of interest.

## A2. Effects of Velositol® on muscular strength, lean mass, whole-body protein balance, and exercise performance during eight weeks of resistance training: Part I

### T. Ziegenfuss^1^, K. Cesareo^1^, M. Carney^1^, C. Kerksick^2^, A. Kedia^1^, J. Sandrock^1^, B. Raub^1^, A. Ferrando^3^, H. Lopez^1^

#### ^1^The Center for Applied Health Sciences, Canfield, OH, 44406, USA; ^2^School of Health Sciences, Lindenwood University, MO, 63301, USA; ^3^The University of Arkansas for Medical Sciences, Little Rock, AR, 72205, USA

##### **Correspondence:** T. Ziegenfuss (TZ@appliedhealthsciences.org)

**Background**

Previously (https://www.ncbi.nlm.nih.gov/pubmed/28194093) we reported that adding a patented complex of chromium picolinate, chromium histidinate and amylopectin (Velositol®) to a single 6 g dose of whey protein increased muscle protein synthesis by 48% vs. a 24% increase from the same dose of protein alone. We aimed to extend these findings by examining chronic changes in muscle strength, fat-free mass (FFM), whole-body protein balance, and exercise performance during eight weeks of resistance training (RT).

**Methods**

Using a randomized, active-controlled, double-blind design, 35 recreationally active men (mean ± age, height, weight: 40.9 ± 7.6 y, 180.2 ± 6.1 cm, 95.8 ± 14.5 kg) were matched according to HOMA-IR and RT experience and then randomly allocated to one of three groups: active group (2 g Velositol**®** + 15 g whey, V15W), comparator group (15 g whey, 15W), or high-dose comparator group (30 g whey, 30W). Subjects consumed their supplement immediately following exercise on training days, and at the same time of day on non-training days. At 0, 4, and 8 weeks, we assessed body composition (4C via DEXA, Bod Pod, Bioimpedance), whole-body protein balance (^15^N-alanine), and upper/lower body performance [1RM and repetitions to failure (RTF) on bench press and squat and vertical jump power]. Note: Biomarker safety and recovery data were also collected and reported separately as “Part II”.

**Results**

All groups gained strength, increased FFM, and improved muscle size. Similarly, all groups increased squat RTF, but the increase for V15W (+25.3 reps) was statistically significant compared to 15W (+12.0 reps) and 30W (+13.9 reps), p=0.02. Similarly, at 4 weeks squat RTF were greater in V15W (+14.4) vs. 15W (+7.8), p=0.02. When performance data were normalized relative to body mass, vertical jump power increased more for V15W (+2.1 W/kg) than either 15W (+0.4 W/kg) or 30W (0.3 W/kg), p=0.03. Relative squat strength also improved more in V15W (+0.33 kg) vs. 30W (+0.22 kg), p=0.05. Vertical jump height increased more in V15W (+8.7 cm) vs. 15W (+1.6 cm) and 30W (+0.9 cm), p=0.04. In the subset (N=15) of men who completed ^15^N-alanine ingestion, net protein balance was significantly greater in V15W compared to 15W and 30W at 4 weeks (p<0.05), but this effect was no longer statistically significant at 8 weeks (p=0.51).

**Conclusions**

Collectively, these findings indicate V15W increases squat strength, RTF, vertical power, and vertical jump height perhaps by optimizing early adaptations in whole-body protein balance.

**Acknowledgements**

This study was funded in part by a research grant from Nutrition 21, LLC. The researchers in this study independently collected, analyzed, and interpreted the results without input from the sponsor. All authors declare no conflict of interest.

## A3. Effects of Velositol® on hemodynamic, hematologic, and biochemical biomarkers of safety and recovery during eight weeks of resistance training: Part II

### H. Lopez^1^, K. Cesareo^1^, M. Carney^1^, A. Kedia^1^, J. Sandrock^1^, B. Raub^1^, C. Kerksick^2^, A. Ferrando^3^, T. Ziegenfuss^1^

#### ^1^The Center for Applied Health Sciences, Canfield, OH, 44406, USA; ^2^School of Health Sciences, Lindenwood University, MO, 63301, USA; ^3^The University of Arkansas for Medical Sciences, Little Rock, AR, 72205, USA

##### **Correspondence:** H. Lopez (HL@appliedhealthsciences.org)

**Background**

Previously (https://www.ncbi.nlm.nih.gov/pubmed/28194093) we reported that the addition of a patented complex of chromium picolinate, chromium histidinate and amylopectin (Velositol®) to a 6 g dose of whey protein increased muscle protein synthesis by 48% vs. a 24% increase from the same dose of protein alone. This study aimed to extend these findings by examining changes in standard hematologic and biochemical biomarkers of safety as well as subjective markers of recovery during eight weeks of resistance training.

**Methods**

Using a randomized, active-controlled, double-blind design, 35 recreationally active men (mean ± age, height, weight: 40.9 ± 7.6 y, 180.2 ± 6.1 cm, 95.8 ± 14.5 kg) were matched according to HOMA-IR [i.e. (fasting glucose x fasting insulin)/405] and resistance-training experience and then randomly allocated to one of three groups: an active group (2 g Velositol**®** + 15 g whey protein, V15W), a comparator group (15 g of whey protein, 15W), or a high-dose comparator group (30 g of whey protein, 30W). Subjects consumed their respective supplement immediately following resistance exercise on training days, and at the same time of day on non-training days. At 0, 4, and 8 weeks of training, measurements of anchored VAS scales for subjective perception of perceived recovery, sleep quality, energy, willingness to train, and muscle soreness were obtained as well as standard hemodynamic, hematologic, and biochemical biomarkers of safety (CBC, lipid panel, fasting insulin and comprehensive metabolic panel).

**Results**

Aside from a statistically significant, yet stochastic interaction for creatinine (p=0.02), all values for hepato-renal function (AST, ALT, BUN, total bilirubin, alkaline phosphatase), fasting blood lipids (cholesterol, triglycerides, HDL, LDL) whole blood cell counts (hemoglobin, hematocrit, RBC, MCV, MCH, MCHC, RDW, differential white cell counts) remained within normal clinical limits, and no between-group differences over time were noted. HOMA-IR, HOMA-B, perceived recovery, and all VAS indices (energy, willingness to exercise, muscle soreness, sleep quality) were also not different between groups. Interestingly, a notable decrease in diastolic BP was noted in V15W (-5.1 mm Hg); this effect was statistically significant compared to the other two groups (p=0.002). Systolic BP also decreased by 5.0 mm Hg in V15W, and although this was a statistically significant within-group effect (p=0.03), the interaction was not significant (p=0.23).

**Conclusions**

Within the confines of this study design, these findings indicate V15W plus eight weeks of RT promotes beneficial decreases in diastolic BP, and is well tolerated relative to standard hematologic and biochemical biomarkers of safety.

**Acknowledgements**

This study was funded in part by a research grant from Nutrition 21, LLC. The researchers in this study independently collected, analyzed, and interpreted the results without input from the sponsor. All authors declare no conflict of interest.

## A4. Divergent respiratory responses between upper and lower body cycling during heavy-intensity isocaloric exercise

### Nicolas W. Clark^1,2^, Valéria L. G. Panissa^3^, David Boffey^1,2^, Jeffrey R. Stout (FISSN)^1,2^, David H. Fukuda^1,2^

#### ^1^School of Kinesiology and Physical Therapy, University of Central Florida, Florida, FL, 32816, USA; ^2^Physiology of Work and Exercise Response (POWER) Laboratory, Institute of Exercise Physiology and Rehabilitation Science, University of Central Florida, FL, 32816, USA; ^3^University of São Paulo, São Paulo, 05508-030, Brazil

##### **Correspondence:** Nicolas W. Clark (nicolas.clark@ucf.edu)

**Background**

Exercise modalities that promote cardiorespiratory endurance while engaging different muscle groups have the potential to extend the potential beneficial impacts of physical activity. There is a paucity of research examining the metabolic responses of upper body cycling (UP) versus lower body cycling (LO) performed at normalized intensities based on the gas exchange threshold (GET). The purpose of this study was to examine the respiratory and metabolic responses to isocaloric sessions of UP and LO at moderate and heavy exercise intensities.

**Material and Methods**

Eight participants (23.1±2.5 years old; 25.1±3.9 kg/m^2^) completed pretesting screening, a familiarization session, and six exercise testing visits. The first two exercise visits consisted of a randomized UP and LO graded exercise test (GXT) to volitional fatigue, in order to determine V̇O_2peak_ and GET. For the remaining four exercise visits, participants completed UP or LO at moderate or heavy exercise intensities. Moderate intensity was calculated as 80% percent of GET [1], and heavy intensity was defined as the work-rate equivalent to 30% of the difference between GET and V̇O_2peak_ [2]. Tests were terminated once participants reached an estimated caloric expenditure of 100 kcal, and the duration of trial was recorded. Respiratory and metabolic variables were evaluated during exercise testing via gas-exchange analysis. A two-way repeated-measures ANOVA [mode(UP vs LO) x intensity (heavy vs. moderate)] with Holm post-hoc tests was used to evaluate the dependent variables.

**Results**

Results are listed in Table 1. A significant interaction (p<0.05) between intensity and mode was shown for tidal volume (VT), inspiratory volume (IV), and VCO_2_. LO/Heavy had significantly higher VT, IV, and VCO_2_ compared to UP/Heavy, but there was no difference between LO/moderate and UP/Moderate. RER was higher for LO compared to UP. For VO_2_ and METS, LO was higher than UP and heavy intensity was higher than moderate intensity. Duration of trials was higher for UP and for moderate trials. Breathing rate (Rf) was significantly different between heavy and moderate intensities, but not between modes.

**Conclusions**

While some metabolic and respiratory differences exist between upper and lower body cycling at heavy exercise intensities, these differences are less pronounced at a moderate exercise intensity. For other variables, such as RER, lower body cycling produced greater responses regardless of exercise intensity. Therefore, exercise modality may also play an important role in substrate oxidation.

**References**

1. Fornasiero A, Skafidas S, Stella F, Zignoli A, Savoldelli A, Rakobowchuk M, et al. Cardiac Autonomic and Physiological Responses to Moderate-Intensity Exercise in Hypoxia. Int J Sports Med. 2019;40(14):886-896.

2. Tiller NB, Campbell IG, Romer LM. Influence of Upper-Body Exercise on the Fatigability of Human Respiratory Muscles: Med Sci Sports Exerc. 2017;49:1461–72.

**Table 1 (abstract A4).** Respiratory and metabolic variables during 100-kilocalorie cycling tests. Values are mean ± SD

	Heavy			Moderate
	Upper body	Lower body	Upper body	Lower body
Trial time (min)^*#^	23.0 ± 2.7	21.0 ± 2.6	28.7 ± 3.4	27.2 ± 5.8
METS^*#^	6.5 ± 1.2	7.7 ± 1.8	4.2 ± 0.8	5.0 ± 1.4
**VO**_**2**_ (mL/kg/min)^*#^	22.6 ± 1.7	27.1 ± 1.7	14.8 ± 1.7	17.3 ± 1.7
**RER**^#^	0.87 ± 0.06	0.96 ± 0.09	0.86 ± 0.08	0.87 ± 0.05
Rf (1/min)^*^	35.3 ± 3.1	30.1 ± 3.1	27.1 ± 3.1	24.4 ± 3.1
**VT L (btps)** ^*#^	1.58 ± 0.51	2.14 ± 0.85^†^	1.27 ± 0.46	1.55 ± 0.56
VE (L/min)^*^	50.7 ± 7.3	59.1 ± 15.7	30.7 ± 4.7	35.0 ± 8.1
**IV (L/min)** ^*#^	1.52 ± 0.50	2.09 ± 0.83^†^	1.20 ± 0.48	1.47 ± 0.58
**VO**_**2**_ (L/min)^*#^	1.73 ± 0.33	2.08 ± 0.52	1.14 ± 0.24	1.35 ± 0.43
**VCO**_**2**_ **(L/min)** ^*#^	1.52 ± 0.34	2.02 ± 0.64^†^	0.98 ± 0.23	1.18 ± 0.39

Note: METS = metabolic equivalents; RER = respiratory exchange ratio; Rf = breathing frequency; VT = tidal volume; VE = exhaled minute ventilation; IV = inspired minute ventilation. *main effect for intensity; ^#^main effect for mode; †significantly different from upper body

## A5. Nutrition education intervention with Major League Rugby team

### Jessica Malone, Katelyn White, Jessie Houston, Laura Zea

#### Department of Nutrition and Basic Sciences, Bastyr University California, San Diego, CA, 92121, USA

##### **Correspondence:** Jessica Malone (jessica.malone@bastyr.edu)

**Background**

Nutrition is key for athletic performance and recovery, making it an important factor for the longevity of an athlete’s career, as well as the success of a professional sports team. Studies have shown that nutrition education can positively impact both nutrition knowledge and nutrition-related behavior change. However, nutrition professionals are not always utilized in professional sports organizations.

**Materials and Methods**

Based on Grounded Theory, a nutrition education intervention was proposed and implemented to investigate this dichotomy. A single cohort, mixed methods population-based study was designed around the specific needs of a Major League Rugby organization’s athletes. The four-week intervention focused on providing nutrition education and behavior change strategies to the participants, who volunteered to participate in the study. Using triangulation research methods, a needs assessment was first conducted to assess participants’ perceptions of nutrition, behaviors, and needs, to be utilized in the development of a nutrition education intervention. Then, quantitative data was collected through the implementation of pre- and post-intervention surveys, in order to evaluate the effects of the intervention in the key areas of nutrition knowledge, self-efficacy in nutrition-related skills, and nutrition-related behavior. Quantitative data was coded by theme and analyzed.

**Results**

Results were based on the inclusion of 11 matching pre-and post-intervention surveys, from a pool of 19 study participants, decreased by the effects of the COVID-19 pandemic. The results showed a small improvement in mean score of nutrition knowledge from 3 to 3.18 out of 5 total points, but this increase was not found to be statistically significant. Self-efficacy by means of confidence in six categories of nutrition-related skills were found to have increased from the pre- to the post-intervention surveys, but were also not found to be statistically significant differences (Table 1). And finally, a weak positive correlation was found between the number of intervention sessions attended and nutrition knowledge score (r = 0.123), but this correlation was not found to be statistically significant. However, upon completion of the intervention, 63.64% of participants self-reported improvement in both nutrition knowledge and behavior, while 81.82% of participants reported adoption of a new nutrition-related behavior.

**Conclusions**

Major findings of this study involve the subjective success of nutrition education intervention with athletes. These findings may be applied to other athletic populations, while adding to the available literature for future research on sports nutrition education intervention.

**Table 1 (abstract A5).** Pre-Survey and Post-Survey Confidence Levels

Confidence category	Percentage of participants in the pre-survey reporting “fairly confident” or “extremely confident”	Percentage of participants in the post-survey reporting “fairly confident” or “extremely confident”	Growth of “fairly confident” or “extremely confident” percentage of participants from pre- to post-survey
How confident are you in your cooking abilities?	73%	82%	9%
How confident are you in your knowledge of nutrition?	64%	91%	27%
How confident are you in your skills when reading a nutrition label?	45%	73%	28%
How confident are you in your skills when grocery shopping for healthy food?	91%	100%	9%
How confident are you in your skills when grocery shopping on a budget?	54%	73%	19%
How confident are you in your ability to put together a balanced meal?	73%	91%	18%

## A6. A comparison between BIA and A-mode ultrasound technology for assessing body composition changes in resistance-trained individuals undergoing short-term calorie restriction

### Jack Quint, Adam Ibrahim, Gianna Mastrofini, Priscila Lamadrid, Brian Waddell, Adriana Gonzales, Lizzie Tarr, Josh Rogers, Daniel Klahr, Kait Callahan, Hunter Miller, Denise Schoucair, Marisa Urrutia, Gillian SanFilippo, Alexander Brooks, Bill I. Campbell

#### Performance and Physique Enhancement Laboratory, University of South Florida, Tampa, FL, 33620, USA

##### **Correspondence:** Bill I. Campbell (bcampbell@usf.edu)

**Background**

The measurement of body composition is of interest to individuals seeking to optimize their physiques. Various methods have been used to assess body composition changes following an exercise or diet intervention. However, more research is needed with respect to comparing different body composition assessment methods. Therefore, the purpose of this study was to compare the differences in body fat % changes between bioelectrical impedance analysis (BIA) and A-mode ultrasound measures in resistance-trained individuals undergoing short-term calorie restriction.

**Materials and Methods**

20 resistance-trained males (n=5) and females (n=15) participated in this study (age: 25.2 ± 7.8 years; height: 167.2 ± 7.9 cm; body mass: 66.6 ± 11.2 kgs). Following a period of baseline caloric tracking, subjects were prescribed a two-week energy-restricted diet (resulting in an approximate 37.5% caloric deficit). Subjects were instructed to ingest 2.2g of protein/kg bodyweight and continue their regular resistance training and aerobic training routine under the supervision of a staff member. Body composition was assessed pre and post-intervention utilizing a BIA and an A-mode ultrasound device. The BIA device used was the InBody® 570 Body Composition Analyzer (Biospace, Inc. Seoul, Korea) and the A-mode ultrasound device used was the BodyMetrix™ Professional A-mode ultrasound (IntelaMetrix, Livermore, CA). Data were analyzed using a dependent samples t-test.

**Results**

No significant differences were observed for the detected changes in body fat percentage between the BIA and A-mode ultrasound devices (p = 0.710). The decrease in body fat percentage after a 2-week caloric deficit was 1.0 ± 0.9% and 1.1 ± 1.0% for the BIA device and A-mode ultrasound, respectively.

**Conclusions**

In resistance-trained individuals, subjects experienced a similar decrease in body fat percentage following a 2-week calorie deficit, whether measured utilizing BIA or A-mode ultrasound technology. Therefore, the current findings indicate that both body composition methods provide equivalent changes in body fat % over time.

## A7. The effect of 2-weeks of intense caloric restriction on resting metabolic rate in resistance-trained individuals

### Alexander Brooks, Madelin R. Siedler, Megan N. Humphries, Sarah Ford, Traci Smith, Sandra Korte, James Gegenheimer, Natalie Fay, Chris Dubie, Shanekque Clarke, Justin Reyes, Olivia Pane, Maria Espinal, David Mathas, Jack Quint, Bill I. Campbell

#### Performance and Physique Enhancement Laboratory, University of South Florida, Tampa, FL, 333620, USA

##### **Correspondence:** Bill I. Campbell (bcampbell@usf.edu)

**Background**

One of the negative consequences of prolonged caloric restriction is a suppression of resting metabolic rate (RMR), making future fat loss more difficult. Further, some evidence suggests that the suppression of RMR is associated with the severity of the caloric deficit (the greater the caloric deficit the greater the reduction in RMR). Strategies employed to prevent the suppression of RMR during a caloric deficit include resistance exercise and a high protein diet. Currently, there is a lack of research investigating the prevalence of metabolic slowing in resistance-training individuals undergoing a short-term, intensive caloric restricted diet that is high in protein. Therefore, the purpose of this study was to examine the effects of short-term intense caloric restriction on resting metabolic rate in resistance-trained males and females eating a relatively high protein diet.

**Materials and Methods**

20 resistance-trained males (n=5) and females (n=15) participated in this study (age: 25.2 ± 7.8 years; height: 167.2 ± 7.9 cm; body mass: 66.6 ± 11.2 kgs). Following a period of baseline caloric tracking, subjects were prescribed 2 weeks of energy restriction resulting in an approximate 37.5% decrease in caloric intake. Subjects were instructed to ingest 2.2 g protein/kg body weight and continue their habitual resistance training routine while dieting. RMR was assessed pre and post-intervention using a Parvo Medics’ TrueOne® 2400 device (ParvoMedics, Sandy, UT). Data were analyzed using a dependent samples t-test.

**Results**

There was a significant decrease in RMR during the 2-week caloric deficit (p = 0.001).

Specifically, RMR decreased from 1,602 ± 211 to 1,508 ± 241 kcals (a decrease of approximately 5.9% during the study intervention).

**Conclusions**

In resistance-trained males and females, a two-week caloric reduction of approximately 37.5% resulted in a significant decline in resting metabolic rate. High protein intake and resistance training were not able to mitigate this decline. As such, to maintain resting metabolic rate during caloric restriction, a short-term intense caloric reduction is not recommended.

## A8. The effect of moderate intermittent versus continuous energy restriction on body composition and resting metabolic rate in resistance-trained females: A randomized controlled trial

### Madelin R. Siedler, Megan N. Humphries, Priscila Lamadrid, Brian Waddell, Sarah Ford, Gillian SanFilippo, Kaitlin Callahan, James Gegenheimer, Justin Reyes, Olivia Pane, Daniel Klahr, Maria Espinal, Marisa Urrutia, Bill I. Campbell

#### Performance and Physique Enhancement Laboratory, University of South Florida, Tampa, FL, 33620, USA

##### **Correspondence:** Bill I. Campbell (bcampbell@usf.edu)

**Background**

Moderate intermittent energy restriction (mIER) entails intermittent, sustained increases in energy intake over the course of a prolonged period of energy restriction. Previous research in men with obesity suggests that mIER may improve the efficiency of fat loss and reduce metabolic adaptations to prolonged energy restriction, while other studies in similar populations show no effect. The purpose of this study was to examine the effects of mIER versus continuous energy restriction in a population of resistance-trained females.

**Materials and Methods**

38 resistance-trained females (age: 22.3 ±4.2 years; height 1.6 ±0.7m) participated in this study. Subjects were randomized to either continuous dieting (CON; n = 18) or intermittent dieting (INT; n = 20). Participants in CON were prescribed 6 weeks of a continuous 25% reduction in energy intake. Participants in INT were prescribed 1 week of energy intake at maintenance levels after every 2 weeks of 25% energy restriction (8 weeks total). Subjects were instructed to ingest 1.8 g protein/kg bodyweight and took part in 3 weekly supervised resistance training sessions. Body weight, body composition, and resting metabolic rate (RMR) was assessed pre and post-intervention. Data were analyzed using a series of linear mixed models with random intercepts.

**Results**

Across all subjects from PRE to POST, there was a mean decrease in body weight (62.7 ±9 kg to 61.5 ±9.2 kg; [p = 0.0002]); percent body fat (25 ±4.4% to 23.6 ±4.8%; [p < 0.0001]), and fat mass (15.9 ±4.6 kg to 14.7 ±4.6kg; [p < 0.0001]). Fat-free mass (46.8 ±5.2 kg to 46.8 ±5.7; p = 0.90) and RMR (1422 ±193 kcal to 1434 ±190 kcal; p = 0.48) did not change from PRE to POST. There were no significant differences between groups for all body composition variables and RMR.

**Conclusions**

In resistance trained females seeking to optimize their physiques, mIER does not improve the efficiency of fat loss and has no effect on FFM and RMR. Incorporating mIER dieting strategies may be employed for those whom desire a short-term break from an energy-restricted diet without fear of impairing fat loss progress.

**Acknowledgements**

Study was funded by the International Scientific Foundation for Fitness and Nutrition

## A9. The effect of moderate intermittent versus continuous energy restriction on body composition on hunger and eating behaviors in resistance-trained females: A randomized controlled trial

### Madelin R. Siedler, Gianna Mastrofini, Alexander Brooks, Jack Quint, Adam Ibrahim, David Mathas, Traci Smith, Natalie Fay, Joshua Rogers, Adriana Gonzalez, Hunter Miller, Elizabeth Tarr, Ecaterina Vasenina, Bill I. Campbell

#### Performance and Physique Enhancement Laboratory, University of South Florida, Tampa, FL, 33620, USA

##### **Correspondence:** Bill I. Campbell (bcampbell@usf.edu)

**Background**

Moderate intermittent energy restriction (mIER) entails intermittent, sustained increases in energy intake over the course of a prolonged period of energy restriction. The purpose of this study (among others) was to examine the effects of mIER versus continuous energy restriction on eating behaviors in a population of resistance-trained females.

**Materials and Methods**

38 resistance-trained females (age: 22.3±4.2 years; height 1.6±0.7m) participated in this study. Subjects were randomized to either continuous dieting (CON; n=18) or intermittent dieting (INT; n=20). Participants in CON were prescribed 6 weeks of a continuous 25% reduction in energy intake. Participants in INT were prescribed 1 week of energy intake at maintenance levels after every 2 weeks of 25% energy restriction (8 weeks total). Subjects were instructed to ingest 1.8 g protein/kg bodyweight and took part in 3 weekly supervised resistance training sessions. At baseline (PRE) and post-intervention (POST), eating behavior was measured via a 51-item three-factor eating questionnaire (TFEQ) which measures an individual’s level of hunger, disinhibition (the loss of control in food intake), and dietary restraint (degree of cognitive control) in daily food intake. Data were analyzed using a series of linear mixed models with random intercepts.

**Results**

Across all subjects, level of dietary restraint did not change from PRE to POST (p = 0.39), nor did level of hunger (p = 0.66). While there were no differences between groups for change over time in these variables (p = 0.20 and p = 0.21, respectively), there was a significant group-by-time interaction for disinhibition (p = 0.03). While no pairwise comparisons were significantly different, a divergent pattern was observed, with mean values for disinhibition generally increasing from PRE to POST in CON (5.17±2.07 to 5.83±1.79; p=0.52) but decreasing from PRE to POST in INT (6.85±3.76 to 6.00±3.58; p=0.26).

**Conclusions**

Though the use of mIER did not appear to affect changes in hunger or dietary restraint over 6 weeks of energy restriction, it may reduce the impact of prolonged energy restriction on measures of disinhibition, potentially helping to promote increased long-term dietary adherence.

**Acknowledgements**

Study was funded by the International Scientific Foundation for Fitness and Nutrition

## A10. Acute ashwagandha supplementation improves cognitive function

### Dante Xing^1^, Choongsung Yoo^1^, Christopher J. Rasmussen^1^, Martin Purpura^2^, Ralf Jäger, FISSN^2^, Richard B. Kreider^1^

#### ^1^Exercise & Sport Nutrition Lab, Human Clinical Research Facility, Texas A&M University, College Station, TX, 77843-4253, USA; ^2^Increnovo LLC, Milwaukee, WI 53202, USA

##### **Correspondence:** Richard B. Kreider (rbkreider@tamu.edu)

**Background**

Chronic supplementation with ashwagandha (*Withania somnifera* root) reduces feelings of stress, improves mood and cognitive function. The potential benefits of acute ashwagandha supplementation on sustained attention, cognitive flexibility, and/or working memory are currently unknown. The purpose of this double-blind, placebo-controlled, crossover study was to examine the effect of acute ingestion of ashwagandha extract on executive function over time.

**Materials and Methods**

13 healthy male and female subjects (24±5 years, 170.0±11.8 cm, 72.9±19.3 kg, 24.8±3.7 kg/m^2^) were randomly assigned to consume 400mg of a proprietary ashwagandha extract (NooGandha®, Specnova Inc., FL) or placebo (PLA). Subjects completed four cognitive function tests (go no-go test, psychomotor vigilance task test, the Berg-Washington card sorting task, and the Sternberg task test), and then ingested a capsule of ashwagandha extract or PLA with 8 ounces of water. Participants repeated cognitive function tests 1, 2, 3, 4, 5, and 6 hours after ingestion of the supplement. After 7 days participants then repeated the experiment while consuming the alternative treatments. Data were analyzed by a General Linear Model multivariate and univariate analyses with repeated measures using body weight as a covariate.

**Results**

Acute supplementation with 400mg ashwagandha extract significantly increased short-term/working memory in one measure of the Sternberg test (letter length 6, present reaction time after 3 and 6 hours). As the list length increases, probe judgments become less accurate and slower, indicating increases in short-term memory and working memory demands. Ashwagandha resulted in sustained attention (maintained reaction times, prevention of mental fatigue) in the Vigilance Task Test, measuring a person's ability to remain heedfully vigilant. In contrast, placebo showed significantly reduced reactions times (task 20, hour 6; overall, hour 3). Ashwagandha increased the ability to both recognize and ‘shift' to a new rule (BCST) compared to baseline, however, change scores showed no beneficial effects, indicating that the difference in the pre-post analysis are due to differences in baseline values and not due to supplementation. Ashwagandha supplementation resulted in faster response times to correctly respond compared to PLA (shows less metal fatigue) in the go/no-go test. However, ashwagandha didn’t seem to alter accuracy relative to the placebo condition, as both treatments decreased the percentage of correct answers.

**Conclusions**

Acute supplementation with 400mg ashwagandha extract resulted in sustained attention and increased short-term/working memory.

**Acknowledgements**

This research was funded by Specnova, Inc.

## A11. No difference between pre-sleep plant or dairy-based protein consumption on peak muscle torque or soreness following morning eccentric exercise in middle-aged men

### H. G. Colannino, P. G. Saracino, M. J. Ormsbee

#### Institute of Sports Sciences and Medicine, Florida State University, Tallahassee, FL, 32306, USA

##### **Correspondence:** M. J. Ormsbee (mormsbee@fsu.edu)

**Background**

The purpose of this study was to evaluate the effectiveness of pre-sleep consumption of plant-based compared to dairy-based protein on muscle recovery after morning eccentric exercise. We hypothesized that the combination of a rice and pea protein consumed pre-sleep would improve muscle recovery to the same degree as a dairy-based protein and both sources would outperform placebo.

**Material and Methods**

In a randomized (stratified by age and body fat percent), double-blind, placebo-controlled design, eighteen middle-aged (age, 40-64 y), recreationally active, men performed an eccentric training protocol (ECC) in the morning. Bilateral knee extension and flexion (ECC) were completed (5 x 15 repetitions) to induce muscle damage. Participants were randomized to consume 40g of either: whey protein isolate (WI, n=6), rice and pea combination (RP, n=6), or a flavor matched, non-caloric placebo (PL, n=6) before sleep, following the morning training session. Meals were provided (15% PRO, 55% CHO, 30% Fat) for five days (two days pre-ECC, the day of ECC, and the following two days during follow up testing) to standardize nutrition. WI, RP, or PL was consumed (40g), 30-minutes pre-sleep. Isometric peak torque (ISOMflex and ISOMext) and subjective muscle soreness were measured using visual analog scales (VAS) at pre-ECC, +24, +48 and +72 hours post-ECC. Data were analyzed with SPSS v25. Outcome variables were measured using mixed RMANOVA and tukey post hoc analysis to determine group differences. All main effects are reported as (F, df_treatment_, df_error_) = F statistic, p-value, partial eta squared (ηp^2^). Significance was set at p < 0.05.

**Results**

There were no significant differences between groups at pre-ECC for any marker. Muscle soreness was significantly elevated from pre-ECC at all timepoints (F_2.418, 36.270_ = 32.952, p = <.001, ηp^2^ = .687), with no differences between groups (F_4.836, 36.270_ = .512, p = .760, ηp^2^ = .064). Peak ISOMext was significantly reduced from pre-ECC at +24-post and +48-post hours returning to pre-ECC at +72hrs-post (F_1.876, 28.140_= 7.364, p = .003, ηp^2^ = .329) with no group differences (F_3.752, 28.140_= 1.012, p = .414, ηp^2^ = .119). Peak ISOMflex was elevated from pre-ECC at all timepoints (F_1.945, 29.169_= 24.017, p = <.001, ηp^2^ = .616) with no differences between groups (F_3.889, 29.169_= .717, p = .584, ηp^2^ = .087).

**Conclusion**

These data suggest that, regardless of the source, pre-sleep protein for 72h did not improve muscle soreness or peak muscle torque following from a morning bout of damaging exercise in middle-aged men.

**Acknowledgements**

This study was funded by Dymatize Nutrition and Milk Specialties Global.

## A12. Creatine and GI distress – fact or fiction?

### Paulina Czartoryski^1^, Haley Watters^1^, Rithin Manimaleth^1^, Jaime Tartar^2^, Jose Garcia^1^, Paige Napolitano^1^, Cailey Weaver^1^, Lia Jiannine,^1^ Corey Peacock^1^ , Jose Antonio^1^

#### ^1^Exercise and Sport Science, NSU Florida, Davie, FL, 33314, USA; ^2^Psychology and Neuroscience, NSU Florida, Davie, FL, 33314, USA

##### **Correspondence:** Jose Antonio (Jose.Antonio@nova.edu)

**Background**

The purpose of this investigation was to determine if creatine affected measures of GI distress.

**Methods**

Twenty-three healthy recreationally active subjects (age: 22±7 years, height: 177±6 centimeters) were randomly assigned to a 4-week treatment that consisted of: 5 grams of creatine (generic), 5 grams of creatine (CreaBev**®**), and control (no creatine). GI symptoms were measured via the SODA assessment (severity of dyspepsia assessment). The “SODA” questionnaire measures in a validated format, perceived gastric upset, or gastric discomfort as well as overall satisfaction for function.

**Results**

There were no differences between groups for any measure of dyspepsia.

**Conclusions**

Creatine (generic or the IP version) has no effect on measures of GI distress.

**Acknowledgements**

We would like to thank Glanbia for providing creatine (generic) and CreaBev**®**.

**Table 1 (abstract A12).** Severity of Dyspepsia Assessment (SODA)

	Pre	Post	Change
**Pain**			
CreaBev**®** n=8	5.5±6.0	7.4±7.1	1.9±6.9
Creatine Control n=8	7.0±7.5	9.5±9.9	2.5±10.1
Control n=7	2.0±0.0	2.0±0.0	0.0±0.0
**Non-Pain**			
CreaBev**®**	10.5±3.9	10.9±2.6	0.4±4.5
Creatine Control	9.0±2.5	10.0±3.3	1.0±2.5
Control	7.9±2.3	8.0±2.6	0.1±0.4
**Satisfaction**			
CreaBev**®**	18.9±5.1	20.6±4.2	1.7±2.9
Creatine Control	21.4±3.5	21.8±3.5	0.4±1.1
Control	23.0±0.0	23.0±0.0	0.0±0.0

Data are expressed as the mean±SD. There were no significant differences (p>0.05) between groups (delta scores)

## A13. Four weeks of creatine supplementation in trained subjects – effects on measures of cognition

### Haley Watters^1^, Rithin Manimaleth^1^, Jaime Tartar^2^, Jose Garcia^1^, Paulina Czartoryski^1^, Paige Napolitano^1^, Jason Curtis^1^, Cailey Weaver^1^, Lia Jiannine,^1^ Corey Peacock^1^ , Jose Antonio^1^

#### ^1^Exercise and Sport Science, NSU Florida, Davie, FL, 33314, USA; ^2^Department of Neuroscience, NSU Florida, Davie, FL, 33314, USA

##### **Correspondence:** Jose Antonio (Jose.Antonio@nova.edu)

**Background**

The purpose of this investigation was to determine if four weeks of daily supplementation of creatine would affect measures of cognition in exercise-trained men.

**Methods**

Twenty-three subjects completed this investigation (age: 22±7 years, body mass: 78±11 kilograms, height: 1.8±0.1 meters). Subjects were randomly assigned to a creatine group (n=16, 5 grams daily) or control (no supplement, n=7). Cognition was assessed via the NIH Toolbox pre and post treatment. Specifically, the following measures were taken: Flanker Inhibitory Control, Dimensional Change Card Sort Test, and Pattern Comparison Processing Speed Test.

**Results**

There were no changes pre versus post for the Flanker Inhibitory Control or the Pattern Comparison Processing Speed Test. However, the creatine group experienced a significant increase in the Dimensional Change Card Sort Test (pre: 103.6±12.9, post: 116.1±13.4, p=0.0017). The control values were as follows (pre: 117.9±12.8, post: 120.1±8.4, p=0.6494).

**Conclusions**

Despite the fact that the creatine group experienced a statistically significant increase in the Dimensional Change Card Sort Test, it should be noted that the baseline values for the control were above average for the age-corrected standard scores (i.e., the normative mean = 100). A t-score of 85 or 115 denotes one standard deviation below or above the normative mean. Inasmuch as the control group was a standard deviation above the normative mean, it makes sense that changes in cognition were a fraction of the changes measured in the creatine group (which started out near the normative mean). We would speculate that creatine supplementation might be beneficial to individuals scoring at or below the normative mean for this measure of cognition.

**Acknowledgements**

We would like to thank Glanbia for providing creatine (generic) and CreaBev®.

## A14. Does creatine supplementation alter total body water?

### Paige Napolitano^1^, Haley Watters^1^, Jonathan Banks^2^, Rithin Manimaleth^1^, Jaime Tartar^2^, Jose Garcia^1^, Paulina Czartoryski^1^, Cailey Weaver^1^, Lia Jiannine,^1^ Corey Peacock^1^ , Alexsandra Beaton^2^, Alexandra Nieto^2^, Catherine Weber^2^, Aysha Patel^2^, Jose Antonio^1^

#### ^1^Exercise and Sport Science, NSU Florida, Davie, FL, 33314, USA; ^2^Psychology and Neuroscience, NSU Florida, Davie, FL, 33314, USA

##### **Correspondence:** Jose Antonio (Jose.Antonio@nova.edu)

**Background**

The purpose of this investigation was to determine if six weeks of daily supplementation of creatine (5 g/d) would affect measures of hydration.

**Methods**

Thirty-eight healthy recreationally active subjects (age: 21±5 years, height: 169±11 centimeters) were randomly assigned to a creatine group (n=19; 9 female, 10 male) that was instructed to consume five grams daily or control (no supplement) (n=19, 10 female, 9 male). Total body water was assessed via the InBody 270. In addition, body composition was determined via dual energy X-ray absorptiometry (DXA). Subjects were instructed to not alter their exercise and diet habits.

**Results**

There were no significant within-group or between-group changes for body mass, bone mineral content (BMC), total body water, fat mass, lean body mass or body fat percentage in either group: body mass (Table 1).

**Conclusions**

In a group of young, healthy male and female subjects, creatine supplementation coupled with no change in training or diet has no effect on total body water; nor does it affect measures of body composition via the DXA.

**Acknowledgements**

We would like to thank Creapure**®** and Dymatize**®** for providing creatine monohydrate.

**Table 1 (abstract A14).** See text for description

	Control Pre	Control Post	Creatine Pre	Creatine Post
Body Mass (kg)	72.6±18.6	73.1±18.4	69.1±15.5	69.3±15.7
Lean body mass (kg)	47.8±13.2	48.7±14.3	47.2±11.9	48.2±12.5
Fat mass (kg)	21.5±9.2	21.0±8.6	18.7±5.9	17.9±5.5
% Body fat	29.8±8.5	29.2±8.6	26.7±6.7	26.3±6.7
BMC (kg)	2.57±0.66	2.58±0.66	2.60±0.50	2.57±0.46
Total body water (L)	38.7±11.2	38.2±11.5	38.7±10.0	39.1±1.4

## A15. An analysis of Anxiety in Professional Mixed Martial Artists and its relation to weight-class

### Tobin Silver^1^, Julian Pino^2^, Jordan Kwamanakweenda^2^, Olivier van Hauwermeiren^2^, Gabriel J. Sanders^3^, Jose Antonio^1^, Corey A. Peacock^1^, Jaime Tartar^2^

#### ^1^Health and Human Performance, NSU Florida, Davie, 33314, FL, USA; ^2^College of Psychology, NSU Florida, Davie, 33314, FL, USA; ^3^Kinesiology, Northern Kentucky University, Highland Heights, 41099, KY, USA

##### **Correspondence:** Jose Antonio (Jose.Antonio@nova.edu)

**Background**

In the last decade, there have been numerous studies that have observed trait anxiety and its various impacts on athletic performance. These studies have analyzed many outcomes due to trait anxiety, including burnout. They have also established a link between increased anxiety and decreased performance. Although research exists demonstrating MMA athletes with less trait anxiety when compared to a control population, limited research exists examining trait anxiety levels between different weight-classes. Therefore, we aim to investigate trait anxiety in professional MMA fighters, between different weight-classes. We hypothesize trait anxiety is higher in heavier weight-classes and lower in lighter weight-classes.

**Methods**

Twenty-six professional MMA (age: 29±7.1 years, body mass: 85.7±12.0 kilograms, height: 181.8±6.9 centimeters, BMI: 25.8±2.3 kg/m^2^) fighters completed a demographic questionnaire and Spielberger Trait Anxiety Scale (STAI) (Trait anxiety section). STAI (positive and negative curiosity, anxiety, depression) was analyzed between weight-class grouping (Group 1: Bantamweight to Featherweight, Group 2: Lightweight to Welterweight, Group 3: Middleweight to Heavyweight). A one-way analysis of variance (ANOVA) was utilized to assess relationships between STAI and groupings.

**Results**

**Conclusions**

In conclusion, the data demonstrates that professional MMA fighters in different weight-classes did not significantly differ on STAI variables, including trait anxiety, trait curiosity, and trait depression.

**Acknowledgements**

N/A

**Table 1 (abstract A15).** STAI

Trait Anxiety Variable	F	P
Trait Anxiety +	0.509	0.608
Trait Curiosity +	0.825	0.451
Trait Depression +	1.594	0.225
Trait Anxiety -	1.264	0.302
Trait Curiosity -	1.129	0.341
Trait Depression -	2.955	0.072

*denotes significance (P≤0.05)

## A16. An analysis of Emotional Self-Regulation in Professional Mixed Martial Artists and its relation to weight class

### Gabriel J. Sanders^1^, Jordan Kwamanakweenda^2^, Olivier van Hauwermeiren^2^, Julian Pino^2^, Corey A. Peacock^3^, Jose Antonio^3^, Tobin Silver^3^, Jaime Tartar^2^

#### ^1^Kinesiology, Northern Kentucky University, Highland Heights, 41099, KY, USA; ^2^College of Psychology, NSU Florida, Davie, 33314, FL, USA; ^3^Dept. Health and Human Performance, NSU Florida, Davie, 33314, FL, USA

##### **Correspondence:** Jose Antonio **(**Jose.Antonio@nova.edu)

**Background**

Mixed Martial Arts (MMA) is a full-combat, weight-categorized sport in which participants are permitted to use a variety of fighting techniques (e.g., kickboxing, BJJ, and judo). Previous research has examined the relationship between emotional self-regulation and athletic performance. Additional research has analyzed self-regulation in MMA fighters when compared to controls. However, limited research has examined emotional self-regulation scores between different weight-classes. Therefore, we aim to investigate the emotional self-regulation in professional MMA based on weight-class groupings.

**Methods**

Twenty-six professional MMA (age: 28±4.1 years, body mass: 85.7±12.0 kilograms, height: 181.8±6.9 centimeters, BMI: 25.8±2.3 kg/m^2^) fighters completed a demographic questionnaire and The Difficulties in Emotional Regulation Scale (DERS-18), an 18-item psychological tool that measures an individual’s overall emotional self-regulation. Subscale scores for the DERS-18 include total score, awareness, clarity, goals, impulse, nonacceptance, and strategies. ANOVA analysis with post hoc independent sample t-tests examined mean differences in DERS-18 scores and professional MMA weight-class groupings (Group 1: Bantamweight to Featherweight, Group 2: Lightweight to Welterweight, Group 3: Middleweight to Heavyweight).

**Results**

**Conclusions**

In conclusion, the data demonstrates that professional MMA fighters in different weight-classes did not significantly differ on DERS-18 Subscales, including total score, awareness, clarity, goals, impulse, nonacceptance, and strategies.

**Acknowledgements**

N/A

**Table 1 (abstract A16).** DERS-18

Subscale	F	P
Total Score	0.311	0.736
Awareness	0.487	0.621
Clarity	0.237	0.791
Goals	1.605	0.223
Impulse	0.111	0.895
Nonacceptance	0.523	0.600
Strategies	0.306	0.739

*denotes significance (P≤0.05)

## A17. Self-Reported training, weight, and mental well-being during COVID-19 lockdown in Professional Mixed Martial Artists

### Corey A. Peacock^1^, Arman Ali^2^, Gabriel J. Sanders^3^, Jose Antonio^1^, Douglas S. Kalman^1^, Tobin Silver^1^, Jaime Tartar^4^

#### ^1^Health and Human Performance, NSU Florida, Davie, FL, 33314, USA; ^2^College of Medicine, NSU Florida, Davie, FL, 33314, USA; ^3^Kinesiology, Northern Kentucky University, Highland Heights, KY, 41099, USA; ^4^College of Psychology, NSU Florida, Davie, FL, 33314, USA

##### **Correspondence:** Jose Antonio (Jose.Antonio@nova.edu)

**Background**

Mixed Martial Arts (MMA) is a weight-restricted, combat sport which combines many disciplines of striking, grappling, and physical preparation. Much research has been done on MMA fighters in terms of training volume, weight-cutting, and psychology, however there is currently a lack of research regarding how COVID-19 lockdown may have influenced each. Therefore, the purpose of this study is to analyze the effects of COVID-19 lockdown in terms of training frequency, weight management, and psychological well-being. We hypothesize that COVID-19 lockdown will result in declines in training frequency, mood state, and weight management.

**Methods**

Seventeen professional MMA fighters (age: 28±4.1 years, body mass: 85.7±12.0 kilograms, height: 181.8±6.9 centimeters, BMI: 25.8±2.3 kg/m^2^) completed a 25 question survey, reporting behaviors associated with mixed martial arts training, nutrition, and mental-wellness. These questions focused on all aspects both pre- COVID-19 lockdown and during-COVID-19 lockdown. Reporting was completed utilizing a nominal scale. Means and measures of variability will be calculated for all descriptive data. A Wilcoxon signed-rank test was utilized to analyze group differences between conditions (pre-, during-).

**Results**

**Conclusions**

The results indicate that COVID-19 lockdown did elicit a statistically significant decrease in self-reported MMA training frequency, grappling training frequency, fight ‘readiness’, and mood state. The results also indicated that COVID-19 lockdown did not elicit a statistically significant change in self-reported strength training frequency, sparring training frequency, striking training frequency, sleep frequency, weight relative to weight class, meal frequency, and anger. These results may support the need for potential physical and mental support following COVID-19 lockdown in professional MMA fighters. Although the fighters were able to maintain weight and meal frequency, they were unable to train adequately to maintain fight ‘readiness.’

**Acknowledgements**

N/A

**Table 1 (abstract A17).** Self-Reported Data

Variable	Z	P
MMA Sessions	-3.317	0.001*
Grappling Sessions	-2.111	0.035*
S&C Sessions	0.707	0.480
Sparring Sessions	-1.667	0.096
Fight Readiness	-2.972	0.003*
Sleep Quantity	-1.000	0.312
Weight relative to Class	-1.000	0.312
Meal Frequency	-1.414	0.157
Mood	-2.121	0.034*
Anger	-1.037	0.102
Happiness	-2.449	0.014*

*denotes significance (P≤0.05)

## A18. An analysis of the Dark Triad Scores in Professional Mixed Martial Artists and its relationship to weight-class

### Jaime Tartar^1^, Olivier van Hauwermeiren^1^, Julian Pino^1^, Jordan Kwamanakweenda^1^, Jose Antonio^2^, Tobin Silver^2^, Gabriel J. Sanders^3^, Corey A. Peacock^2^

#### ^1^College of Psychology, NSU Florida, Davie, 33314, FL, USA; ^2^Exercise and Sports Science, NSU Florida, Davie, 33314, FL, USA; ^3^Kinesiology, Northern Kentucky University, Highland Heights, 41099, KY, USA

##### **Correspondence:** Jose Antonio (Jose.Antonio@nova.edu)

**Background**

The Dark Triad is composed of three personality traits including narcissism, psychopathy, and Machiavellianism. Prior research of the Dark Triad has analyzed trait differences between men and women, veteran athletes and rookie athletes, and mixed martial artists (MMA) and college-controls. Psychopathy has also been linked to performance in MMA bout performance. Although MMA research exists in regards to the Dark Triad, limited research exists analyzing the personality traits of the Dark Triad in regards to different MMA weight-classes. Therefore, we aim to investigate the Dark Triad in professional MMA based on physical size between different weight-class groupings. We hypothesize that differences will exist between different weight-classes.

**Methods**

Twenty-six professional MMA (age: 28±4.1 years, body mass: 85.7±12.0 kilograms, height: 181.8±6.9 centimeters, BMI: 25.8±2.3 kg/m^2^) fighters completed the Short Dark Triad, a brief version of the Dark Triad questionnaire. A one-way analysis of variance (ANOVA) was utilized to assess relationships between Short Dark Triad subscale scores (narcissism, psychopathy, and Machiavellianism) and weight-class grouping (Bantamweight to Featherweight, Lightweight to Welterweight, Middleweight to Heavyweight).

**Results**

**Conclusions**

In conclusion, the data demonstrates that professional MMA fighters in different weight-classes did not significantly differ on Dark Triad Subscales, including Machiavellianism, Narcissism, and Psychopathy.

**Acknowledgements**

N/A

**Table 1 (abstract A18).** Short Dark Triad

Personality Trait	F	P
Machiavellianism	0.927	0.410
Narcissism	2.044	0.152
Psychopathy	0.237	0.791

*denotes significance (P≤0.05)

## A19. The Effects of Bang Energy Drink on Sexual Functioning

### Lia Jiannine, Lorena Hernandez, Jose Antonio

#### Exercise and Sport Science, NSU Florida, Davie, FL, 33314, USA

##### **Correspondence:** Jose Antonio (Jose.Antonio@nova.edu)

**Background**

The purpose of this investigation was to determine if the acute consumption of an energy drink affected sexual functioning in men and women.

**Methods**

Eight subjects received 6 cans (3 Bang® energy drink and 3 placebo) and were told consume either an energy drink or a placebo 60 minutes before sexual activity. Both consumers and their partners filled out an online questionnaire within 30 minutes of sexual activity assessing orgasm, satisfaction, duration, enjoyment and intensity, pleasure and perceived sexual attractiveness. An unequal variance t- test was performed.

**Results**

Based on this limited sample, Bang® energy drink appears to influence self-reported sexual enjoyment of male consumers (p = 0.046) and sexual satisfaction female consumers (p=0.031). Male partners reported significant higher levels of satisfaction (p =0.049) and enjoyment (p =0.044). Female partners reported significantly higher levels of intensity (p=0.035) and control (p =0.044)

**Conclusions**

Based on this limited sample, it is possible that a caffeine-containing energy drink might alter the perception of sexual satisfaction in both men and women.

**Acknowledgements**

We would like to thank Bang® for providing the energy drinks.

## A20. Does the acute consumption of water or a protein shake affect body composition measures via the InBody270?

### Lia Jiannine, Jose Antonio

#### Exercise and Sport Science, NSU Florida, Davie, FL, 33314, USA

##### **Correspondence:** Jose Antonio (Jose.Antonio@nova.edu)

**Background**

The purpose of this study was to examine the effects of consuming either 0.6 liters of water or an isovolumic protein shake (160 kcal, 3 g fat, 4 g carbohydrate, 30 g protein) on indices of body composition.

**Methods**

Forty-two recreationally active men (n=13) and women (n=29) (mean±SD: 168±10 cm, 22±5 yr, 69.8±11.2 kg) consumed 0.6 liters of water or a protein shake (160 kcal, 3 g fat, 4 g carbohydrate, 30 g protein) in a randomized, counterbalanced fashion. Body composition was assessed via multi-frequency bioelectrical impedance (InBody 270) at baseline, immediately post-consumption (0 minutes), 30 minutes post-consumption, and 60 minutes post-consumption.

**Results**

There were no significant differences between baseline and any time point for the water and protein condition for body mass, lean body mass, and fat mass. Under the protein treatment, there were no significant differences between baseline or any time point for total body water; however, significant differences were found under the water condition (time point 0 and 60 minutes were significantly lower versus baseline). Under both the water and protein conditions, percent body fat was significantly greater (p<0.0001) at time points 0, 30 and 60 minutes compared to baseline (Table 1 and 2).

**Conclusions**

The acute consumption of either water or an isovolumic protein shake resulted in a measurable increase in percent body fat immediately post-consumption as well as 30 and 60 minutes thereafter.

**Table 1 (abstract A20).** Body Composition Post-Consumption of Water

	Baseline	0 min	30 min	60 min
Body Mass (kg)	69.8±11.2	70.4±11.2	70.3±11.2	70.3±11.2
Lean Body Mass (kg)	52.7±11.1	52.5±11.2	52.6±11.2	52.4±11.2
Fat Mass (kg)	17.1±6.6	17.8±6.6	17.7±6.7	17.9±6.8
Total Body Water (Liters)	38.6±8.1	38.4±8.2	38.5±8.2	38.3±8.2
Percent Body Fat	24.6±8.9	25.5±8.8*	25.3±8.9*	25.6±9.0*

Data are presented as the mean±SD. Legend: kg (kilogram); min (minute)

*p<0.0001 versus baseline

**Table 2 (abstract A20).** Body Composition Post-Consumption of Protein

	Baseline	0 min	30 min	60 min
Body Mass (kg)	69.3±11.1	69.9±11.1	69.9±11.1	69.8±11.1
Lean Body Mass (kg)	52.4±11.0	52.4±11.0	52.3±11.0	52.4±11.0
Fat Mass (kg)	16.9±6.8	17.5±6.7	17.6±6.8	17.5±6.8
Total Body Water (Liters)	38.4±8.0	38.3±8.0	38.0±8.4	38.3±8.0
Percent Body Fat	24.5±9.1	25.2±9.0*	25.3±9.1*	25.1±9.1*

Data are presented as the mean±SD. Legend: kg (kilogram); min (minute)

*p<0.0001 versus baseline

## A21. The impact of creatine on cognitive functioning: Does exercise frequency matter?

### Catherine Weber^1^, Alexsandra Alvarez-Beaton^1^, Aysha Patel^1^, Paige Napolitano^2^, Haley Watters^2^, Jose Garcia^2^, Rithin Manimaleth^2^, Jose Antonio^2^, & Jonathan B. Banks^1^

#### ^1^Department of Psychology and Neuroscience; Nova Southeastern University, Davie, FL, 33314, USA; ^2^Department of Health and Human Performance, Nova Southeastern University, Davie, 33314, FL, USA

##### **Correspondence:** Jonathan B. Banks (jonathan.banks@nova.edu)

**Background**

Creatine is an extremely popular nutritional aid used by athletes due to the well-established benefits including increased tolerance to heat [1] and increased performance on high intensity exercise [2, 3]. Of interest to the current study are possible benefits to cognitive functioning. Creatine appears to protect cognitive functioning following sleep deprivation [4], in the face of boredom [5] and on demanding executive functioning tasks [4]. The purpose of the current study was to examine how exercise frequency may alter the impact of a 6-week creatine supplementation on cognitive functioning.

**Methods**

49 undergraduate students were assigned to either a creatine condition (*n*=25), in which they consumed 5 grams of creatine per day for a 6-week period, or a wait-list control condition (*n*=24), in which they did not consume creatine. Subjects completed measures of working memory, sustained attention, speed of processing, and mind wandering and measures of body composition via the dual-energy x-ray absorptiometry (DXA) prior to and at the end of the 6-week period. Subjects indicated their frequency of exercise on a demographics form.

**Results**

We conducted a linear- mixed model regression analysis predicting working memory, sustained attention, mind wandering, and speed of processing from condition, time, and the interaction between condition and time. Analyses were conducted separately for individuals that reported exercising frequently (e.g. more than 3 times per week, *n* = 16) and those that reported exercising infrequently (e.g. less than 3 times per week, *n* = 32). We did not find a significant interaction between time and condition when predicting working memory, sustained attention, mind wandering, or speed of processing for frequent exercisers. For infrequent exercisers, we observed significant time by condition interactions predicting dprime (a measure of sustained attention), b = 0.72, *p* = .027, mind wandering, b = -0.22, *p* = .022, and reaction time variability, b = -52.84, *p* = .024. For all of these measures, improvements in performance were observed in the creatine condition but not the control condition for infrequent exercisers.

**Conclusions**

The current results suggest that creatine supplementation may improve sustained attention performance and reduce mind wandering in young healthy adults, but the effect appears to occur for individuals that do not engage in regular exercise. Future studies should examine how type of exercise may alter these findings.

**Acknowledgements**

We would like to thank Dymatize and Creapure for providing the creatine monohydrate used in this investigation.

**References**

1. Kilduff LP, Georgiades E, James N, Minnion RH, Mitchell M, Kingsmore D, Hadjicharlambous M, Pitsiladis YP. The effects of creatine supplementation on cardiovascular, metabolic, and thermoregulatory responses during exercise in the heat in endurance-trained humans. International Journal of Sport Nutrition & Exercise Metabolism. 2004 Aug 1;14(4).

2. Cornish SM, Chilibeck PD, Burke DG. The effect of creatine monohydrate supplementation on sprint skating in ice-hockey players. Journal of Sports Medicine and Physical Fitness. 2006 Mar 1;46(1):90.

3. Dawson B, Vladich TO, Blanksby BA. Effects of 4 weeks of creatine supplementation in junior swimmers on freestyle sprint and swim bench performance. Journal of strength and conditioning research. 2002 Nov;16(4):485-90.

4. McMorris T, Harris RC, Swain J, Corbett J, Collard K, Dyson RJ, Dye L, Hodgson C, Draper N. Effect of creatine supplementation and sleep deprivation, with mild exercise, on cognitive and psychomotor performance, mood state, and plasma concentrations of catecholamines and cortisol. Psychopharmacology. 2006 Mar 1;185(1):93-103.

5. Watanabe A, Kato N, Kato T. Effects of creatine on mental fatigue and cerebral hemoglobin oxygenation. Neuroscience research. 2002 Apr 1;42(4):279-85.

## A22. A double-blinded, placebo-controlled trial evaluating the anti-inflammatory effects of Curcugen^TM^ in an acute exercise model

### Neil A. Schwarz^1^, Sarah K. McKinley-Barnard^1^, Sri Prahadeeswaran^1^, Jeffrey S. Martin^2^

#### ^1^University of South Alabama, Mobile, AL, 36688, USA; ^2^Debusk College of Osteopathic Medicine at Lincoln Memorial University-Knoxville, Knoxville, TN, 37752, USA

##### **Correspondence:** Neil A. Schwarz **(**neilschwarz@southalabama.edu)

**Background**

We sought to evaluate the effect of a water-dispersible curcumin supplement, Curcugen® (CUR; DolCas-Biotech, LLC., Landing, NJ) on select outcomes associated with damaging exercise. We hypothesized that Curcugen® ingestion prior to a bout of intense exercise and during subsequent recovery days would improve outcomes associated with performance, joint range-of-motion (ROM), soreness, and markers of inflammation and oxidative stress when compared with placebo (PLA) supplementation.

**Materials and Methods**

Twenty-four participants were block-randomized to one of two groups, PLA [n=12 (6 M/6 F); age: 22.2±3.3 yrs, BMI: 23.4±2.9 kg/m^2^] or CUR [n=12 (6 M/6 F); age: 21.0±2.4 yrs, BMI: 24.8±2.9 kg/m^2^]. Participants refrained from exercise for 72-h and nutritional supplements for ≥2 weeks prior to participation. At baseline, the following were performed: venipuncture, pressure-to-pain threshold (PPT) soreness assessment of the *vastus lateralis* (VL) and *gastrocnemius* (GC), knee ROM, and 3 trials of countermovement and squat jumps on a force plate. Thereafter, participants consumed a 500 mg PLA (rice flour) or 500 mg CUR capsule with water, rested for 45-min, and then completed an exercise protocol (50 jumps over 50 cm hurdles and 50 drop jumps from a 50 cm plyometric box). Immediately post-exercise jump performance was re-assessed. Venipuncture, PPT, knee ROM, and additional jump performance assessments were also performed at 1-h, 24-h, 48-h, and 72-h post-exercise. PLA and CUR groups consumed 500mg of their respective supplement 2-h prior to the 24-h, 48-h, and 72-h time points. Blood was analyzed for high-sensitivity C-reactive protein (hsCRP), protein carbonyls (PC) and myoglobin (MYO). Data were analyzed between-groups at each post-exercise timepoint via independent t-tests on change scores from baseline.

**Results**

No differences were observed between groups for jump performance. Change in knee ROM from baseline was significantly greater (*p*=0.01) for CUR (+6.8 ± 8.9°) at 72-h post-exercise compared to PLA (-6.3 ± 14.1°). Change in PPT for the proximal VL from baseline was significantly greater for CUR at 48-h (CUR= +12.7 ± 20.6 N, PLA= -2.6 ± 10.8 N; *p*=0.04) and 72-h (CUR= +10.3 ± 16.3 N, PLA= -5.3 ± 14.7 N; *p*=0.02) post-exercise compared to PLA. Change in hsCRP from baseline was different for CUR at 1-h (CUR= -226.7 ± 233.6 ng/mL, PLA= -202.3 ± 457.0 ng/mL; *p*=0.01) and 24-h (CUR= -107.4 ± 535.8 ng/mL, PLA= +418.1 ± 525.7 ng/mL; *p*=0.02) post-exercise compared to PLA. Change in PC from baseline was different for CUR at 24-h (CUR= -5.1 ± 6.4 nmol/mL, PLA= +0.8 ± 6.0 nmol/mL; *p*=0.04) post-exercise compared to PLA. No difference in changes from baseline between groups was observed for MYO illustrating similar muscle damage between groups.

**Conclusions**

Curcugen® may provide anti-inflammatory, anti-oxidant and analgesic effects following acute exercise at a daily dose of 500 mg.

**Acknowledgements**

This study was supported through research funding provided by DolCas-Biotech, LLC to JS Martin and NA Schwarz.

