# Correction to: The effect of moderate intermittent versus continuous energy restriction on body composition and resting metabolic rate in resistance-trained females: A randomized controlled trial

**DOI:** 10.1186/s12970-020-00402-4

**Published:** 2020-12-29

**Authors:** Madelin R. Siedler, Eric T. Trexler, Megan N. Humphries, Priscila Lamadrid, Brian Waddell, Sarah Ford, Gillian SanFilippo, Kaitlin Callahan, James Gegenheimer, Justin Reyes, Olivia Pane, Daniel Klahr, Maria Espinal, Marisa Urrutia, Bill I. Campbell

**Affiliations:** Performance and Physique Enhancement Laboratory, University of South Florida, Tampa, FL 33620 USA

**Correction to: J Int Soc Sports Nutr 17, 59 (2020)**

**https://doi.org/10.1186/s12970-020-00382-5**

Following publication of the supplement [1], the author reported that the 2nd author was omitted from the author group. Eric T. Trexler has been added to the author group and is presented correctly in this correction article.

The supplement [1] has been updated.

**A8.**

**The effect of moderate intermittent versus continuous energy restriction on body composition and resting metabolic rate in resistance-trained females: A randomized controlled trial**

Madelin R. Siedler, Eric T. Trexler, Megan N. Humphries, Priscila Lamadrid, Brian Waddell, Sarah Ford, Gillian SanFilippo, Kaitlin Callahan, James Gegenheimer, Justin Reyes, Olivia Pane, Daniel Klahr, Maria Espinal, Marisa Urrutia, Bill I. Campbell Performance and Physique Enhancement Laboratory, University of South Florida, Tampa, FL, 33620, USA

**Correspondence:** Bill I. Campbell (bcampbell@usf.edu)

Journal of the International Society of Sports Nutrition 2020, 17(Suppl 2):A8.

**Background**

Moderate intermittent energy restriction (mIER) entails intermittent, sustained increases in energy intake over the course of a prolonged period of energy restriction. Previous research in men with obesity suggests that mIER may improve the efficiency of fat loss and reduce metabolic adaptations to prolonged energy restriction, while other studies in similar populations show no effect. The purpose of this study was to examine the effects of mIER versus continuous energy restriction in a population of resistance-trained females.

**Materials and Methods**

38 resistance-trained females (age: 22.3 ±4.2 years; height 1.6 ± 0.7m) participated in this study. Subjects were randomized to either continuous dieting (CON; n = 18) or intermittent dieting (INT; n = 20). Participants in CON were prescribed 6 weeks of a continuous 25% reduction in energy intake. Participants in INT were prescribed 1 week of energy intake at maintenance levels after every 2 weeks of 25% energy restriction (8 weeks total). Subjects were instructed to ingest 1.8 g protein/kg bodyweight and took part in 3 weekly supervised resistance training sessions. Body weight, body composition, and resting metabolic rate (RMR) was assessed pre and post-intervention. Data were analyzed using a series of linear mixed models with random intercepts.

**Results**

Across all subjects from PRE to POST, there was a mean decrease in body weight (62.7 ±9 kg to 61.5 ±9.2 kg; [p = 0.0002]); percent body fat (25 ±4.4% to 23.6 ±4.8%; [p < 0.0001]), and fat mass (15.9 ±4.6 kg to 14.7 ±4.6kg; [p < 0.0001]). Fat-free mass (46.8 ±5.2 kg to 46.8 ±5.7; p = 0.90) and RMR (1422 ±193 kcal to 1434 ±190 kcal; p = 0.48) did not change from PRE to POST. There were no significant differences between groups for all body composition variables and RMR.

**Conclusions**

In resistance trained females seeking to optimize their physiques, mIER does not improve the efficiency of fat loss and has no effect on FFM and RMR. Incorporating mIER dieting strategies may be employed for those whom desire a short-term break from an energy-restricted diet without fear of impairing fat loss progress.

**Acknowledgements**

Study was funded by the International Scientific Foundation for Fitness and Nutrition.
